# Electrogenic transport and K^+^ ion channel expression by the human endolymphatic sac epithelium

**DOI:** 10.1038/srep18110

**Published:** 2015-12-14

**Authors:** Sung Huhn Kim, Bo Gyung Kim, Jin Young Kim, Kyung Jin Roh, Michelle J. Suh, JinSei Jung, In Seok Moon, Sung K. Moon, Jae Young Choi

**Affiliations:** 1 Department of Otorhinolaryngology, Yonsei University College of Medicine, 50-1 Yonsei-Ro, Seodaemun-Gu, Seoul, 03722, Republic of Korea; 2 The Airway Mucus Institute, Yonsei University College of Medicine, 50-1 Yonsei-Ro, Seodaemun-Gu, Seoul, 03722, Republic of Korea; 3 Department of Otorhinolaryngology, Sooncheonhyang University, College of Medicine, 170 Jomaru-ro, Wonmi-Gu, Bucheon City, Gyenggi-Do, 14584, Republic of Korea; 4 Research Center for the Human Natural Defense System, Yonsei University College of Medicine, 50-1 Yonsei-Ro, Seodaemun-Gu, Seoul, 03722, Republic of Korea; 5 Pathogenesis of Ear Diseases Laboratory, Department of Head and Neck Surgery, David Geffen School of Medicine, University of California Los Angeles, 2100 W. Third St, Los Angeles, CA, 90057, USA

## Abstract

The endolymphatic sac (ES) is a cystic organ that is a part of the inner ear and is connected to the cochlea and vestibule. The ES is thought to be involved in inner ear ion homeostasis and fluid volume regulation for the maintenance of hearing and balance function. Many ion channels, transporters, and exchangers have been identified in the ES luminal epithelium, mainly in animal studies, but there has been no functional study investigating ion transport using human ES tissue. We designed the first functional experiments on electrogenic transport in human ES and investigated the contribution of K^+^ channels in the electrogenic transport, which has been rarely identified, even in animal studies, using electrophysiological/pharmacological and molecular biological methods. As a result, we identified functional and molecular evidence for the essential participation of K^+^ channels in the electrogenic transport of human ES epithelium. The identified K^+^ channels involved in the electrogenic transport were KCNN2, KCNJ14, KCNK2, and KCNK6, and the K^+^ transports via those channels are thought to play an important role in the maintenance of the unique ionic milieu of the inner ear fluid.

The inner ear is composed of three luminal structures termed the cochlea, vestibule and endolymphatic sac (ES) (Fig. 1); the main roles of the inner ear are detecting sound and angular/linear acceleration stimulation, which occur in the cochlear and vestibular systems, respectively. The outer border of the membranous inner ear is lined with various types of epithelial cells, and the luminal space is filled with a fluid called endolymph, which has a unique ion composition (high [K^+^] and low [Na^+^] in the cochlea and vestibule and high [Na^+^] and low [K^+^] in the ES, Fig. 1)^1^,^2^,^3^,^4^. This unique ion composition is important for the maintenance of normal hearing and balance because it provides K^+^ into sensory hair cells of the cochlea and vestibule, which depolarizes the sensory epithelium during sound and acceleration stimuli. Among the three structures, the ES is a cystic organ that is situated on the posterior fossa dura and is connected to the cochlear and vestibular labyrinth through the ductus reuniens, the utricular duct, and the saccular duct (Fig. 1). The luminal surface of the ES is covered with a single layer of epithelium that is composed of two main cell types: mitochondria-rich cells (light cells) and ribosome-rich cells (dark cells)^5^,^6^. The mitochondria-rich cells contain various ion channels and transporters on their membranes and are thought to be mainly involved in the ion homeostasis of the luminal fluid of the inner ear, whereas the ribosome-rich cells contain many secretory granules and are thought to be involved in protein synthesis and secretion^6^,^7^,^8^,^9^,^10^. A disconnection between ES and the cochleo-vestibular compartment by ligating the endolymphatic duct increases the endolymphatic fluid volume (endolymphatic hydrops) in guinea pig^11^. Based on the suggested cellular functions and animal experimentation, one of the possible roles of ES has been thought to be the regulation of inner ear ion homeostasis and endolymph volume. However, the luminal fluid of the ES has different ion and protein compositions (high Na^+^, low K^+^, and high protein concentration, Fig. 1) compared to the endolymph of the cochlear and vestibular compartment^1^,^2^,^3^,^4^, and the mechanism of the ES in the regulation of ion homeostasis and fluid volume of the whole inner ear remains unknown.

It has been thought that various ion transport activities in ES play an important role in regulating ion homeostasis of the inner ear; however, there have been no reports of a functional experiment for ion transport in human ES epithelium. Therefore, we attempted to investigate the electrogenic transport occurring in human endolymphatic sac epithelium, as it had not been reported elsewhere. Various ion channels, transporters, and exchangers have been reported in ES epithelium only by animal or cultured cell experiments: a highly Na^+^-permeable non-selective cation channel, Na^+^-Cl^−^ co-transporter, Na^+^-K^+^-2Cl^−^ co-transporter (NKCC), epithelial Na^+^ channel (ENaC), Na^+^-H^+^ exchanger (NHE), pendrin, and H^+^-ATPase on the apical side and Na^+^, K^+^-ATPase and K^+^ channels on the basolateral side^12^. The distribution of these ion channels, transporters, and exchangers according to specific epithelial cell types was not functionally identified because of difficulty in the identification of cell types during functional experiments. In particular, there are several limitations in the functional studies of ion transport in human ES epithelium. First, opportunities for obtaining human ES tissue are rare. However, as harvesting ES can deteriorate inner ear function, the tissue is best obtained from human subjects during inner ear function–destructive surgery, such as acoustic tumour removal. Second, cell types of human ES are more varied than are those of animals, and it is difficult to definitely divide them into the two cell types^13^. Therefore, it is very difficult to identify the specific cell types during functional studies.

In this study, we measured the net physiologic trans-epithelial current that is produced by various ion channels in the human ES epithelium harvested during acoustic tumour surgery instead of measuring the current that is produced by a single cell because the specific cell types could not be identified during the measurement, and the net trans-epithelial current is more indicative of whole-organ function. During the experiment, we found that K^+^ channels essentially participated in the electrogenic transport in human ES epithelium in almost all cases, a phenomenon that had not been directly identified in animal or human ES tissues. Therefore, we tried to investigate certain K^+^ channels and exchangers that contribute to electrogenic transport in human ES using not only electrophysiological and pharmacological methods but also molecular biological methods, such as RT-PCR and immunohistochemistry. The results of this study may contribute to the elucidation of the role of human ES in the regulation of inner ear ion homeostasis, which is essential for maintaining normal hearing and balance functions.

## Results

### Net trans-epithelial ion transport current

**Figure 2:**
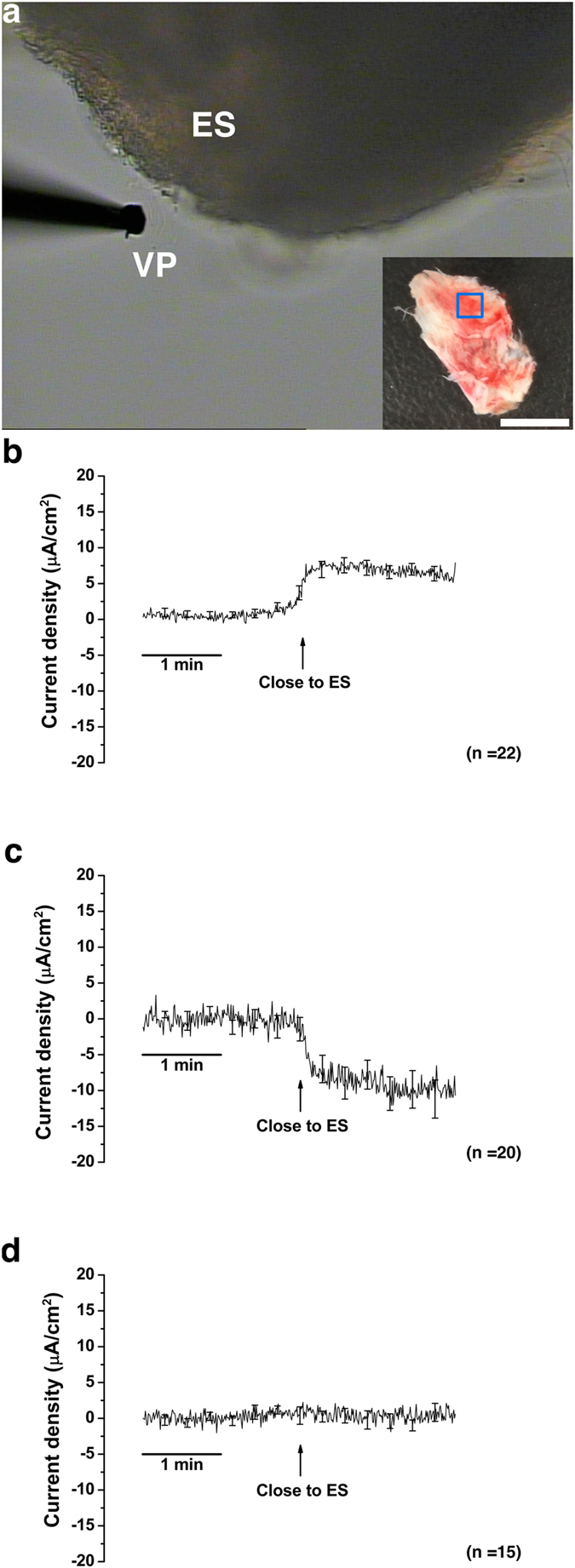
Different net current vectors that were detected during the measurement of the trans-epithelial current from the human endolymphatic sac (ES) epithelium. (**a**) A picture taken during trans-epithelial current measurement (×100). ES was excised into ~ 3 × 3-mm-sized pieces (blue rectangle in the whole human ES sample in smaller rectangular figure. Scale bar = 5 mm) and smoothly folded before measurement. The trans-epithelial current was measured with a vibrating probe (VP) located at a distance of 5 ± 2 μm from the ES epithelium. (**b**) Net current vector of cation secretion/anion absorption (type 1). (**c**) Net current vector of cation absorption/anion secretion (type 2). (**d**) Net neutral current vector (type 3). The averages of all of the experiments are shown; SE is drawn only at intervals for clarity.

We measured the trans-epithelial current from the human ES epithelium of the tissue sample which had been excised into approximately 3 × 3 mm and then smoothly folded apical side-out using the scanning vibrating electrode technique (Fig. 2a). When the electrode was moved from the reference position (400 μm from the tissue) close to the human ES epithelium, three types of current vectors were observed (Fig. 2b-c). The net-transepithelial current vector could be classified into three types: type 1 - cation secretion/anion absorption current vector [mean: 6.4 ± 0.1 μA/cm^2^ (*p* < 0.001), n = 22]; type 2 - cation absorption/anion secretion current vector [mean: -10.0 ± 0.3 μA/cm^2^ (*p* < 0.001) n = 20]; and type 3 - neutral current vector [no detectable current change compared to the current from the reference position; mean: 0.2 ± 0.2 μA/cm^2^ (*p* > 0.05), n = 15]. The current vectors were randomly changed according to the location of the measurement. This result implies that ion transport in the human ES epithelium was so heterogeneous that the net current vector could be different depending on the area of measurement.

### Trans-epithelial transport current

**Figure 3:**
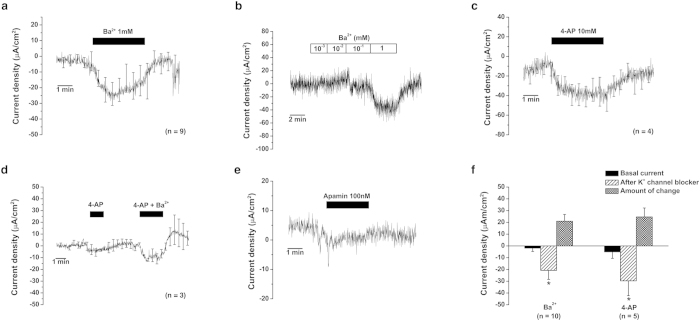
Changes in the trans-epithelial current from human ES after the application of K^+^ channel blockers. (**a**) Changes of trans-epithelial current after Ba^2+^ (1 mM) application. (**b**) Dose-dependent changes of trans-epithelial current after various concentrations of Ba^2+^. (**c**) Inhibition of the K^+^ secretion current after 4-AP (10 mM) application. (**d**) Larger effect of the simultaneous application of 4-AP (10 mM) and Ba^2+^ (1 mM) than that of 4-AP (10 mM) alone on the trans-epithelial current. (**e**) Representative figure for the changes of trans-epithelial current after apamin (100 nM) application. (**f**) Comparison of the mean basal current, changes in the current after the application of K^+^ channel blockers, and the absolute amount of current density change according to the K^+^ blockers that were used. The changes in the current density after the application of Ba^2+^ (1 mM) and 4-AP (10 mM) were greater than the amount of basal current. Values are means ± SE. All figures except for Figures b and e show the averages of all experiments; SE is drawn only at intervals for clarity in Figures a, c, and d. *p < 0.05.

During the experiment using pharmacologic agents to block various ion channels, transporters, and exchangers, we found that the trans-epithelial current likely mediated by K^+^ ions was most consistently detected. When we applied Ba^2+^ (1 mM), a wide-spectrum non-specific K^+^ channel blocker, the cation-secretion current decreased in 9 of 10 cases (amount of mean current change: 18.9 ± 5.5 μA/cm^2^, n = 10, *p* = 0.032, Fig. 3a,f): 4 cases with type 1 basal current vector, 2 cases with type 2 basal current vector, and 3 cases with type 3 basal current vector. No significant current change was detected even after Ba^2+^ application in one case with type 3 basal current vector. The amounts of current change after Ba^2+^ application were significantly larger than the amounts of basal current in all cases (Fig. 3f). The current change was insignificant with Ba^2+^ concentrations of 10^−3^ and 10^−2^ mM; however, the current was significantly changed at a Ba^2+^ concentration of 10^−1^ mM and changed more in terms of magnitude at a concentration of 1 mM (Fig. 3b). These findings were most similar to the characteristics of KCNJ13, KCNJ14, KCNK2, and KCNK6^14^,^15^.

When the non-specific K^+^ channel blocker 4-aminopyridine (4-AP, 10 mM), which mostly blocks voltage-gated K^+^ channels, was applied, the cation secretion current significantly decreased in 4 of 5 cases (mean current change: 24.6 ± 7.6 μA/cm^2^, n = 5, *p* = 0.049, Fig. 3c,f): 1 case with type 1 basal current vector, and 3 cases with type 2 basal current vector. The amount of current changes after the application of 4-AP was also larger than the amount of basal current (Fig. 3f). The transepithelial current was not significantly changed after 4-AP application in 1 case with type 2 basal current vector. The simultaneous application of 4-AP and Ba^2+^ decreased the cation secretion current more than did the application of 4-AP only in 3 cases with type 3 basal current vectors (amount of mean current change after 4-AP application and 4-AP + Ba^2+^ application was 4.9 ± 0.2 μA/cm^2^ and 11.0 ± 2.1 μA/cm^2^, respectively, *p* = 0.037, Fig. 3d).

We tried to apply apamin (100 nM), a specific SK channel blocker, because an apamin-sensitive SK channel (KCNN2 channel) was later identified in liquid chromatography tandem mass spectrometry (LC-MS/MS; see section *Ion channels, transporters, and exchangers in human ES as identified by LC-MS/MS*). After the application of apamin, the cation-secretion current decreased in 2 cases with type 1 basal current vectors (current change: 3.9 and 2.1 μA/cm^2^ for each respective experiment, Fig. 3e) and the amount of current changes was not larger than the amount of basal current.

In summary, the amount of mean current changes was larger than the amount of mean basal current after the application of Ba^2+^ and 4-AP (Fig. 3f) and was not larger than the amount of mean basal current after the application of apamin. The larger amount of current changes after the application of Ba^2+^ and 4-AP than the amount of basal current represents the presence of multiple ion transports with opposite current vectors (cation absorption/anion secretion and cation secretion/anion absorption), and the K^+^ channel blockers inhibited apical secretion of K^+^ channels or influenced the apical cation secretion/anion absorption through multiple ion channels by inhibiting K^+^ channel activities in human ES epithelium.

**Figure 4:**
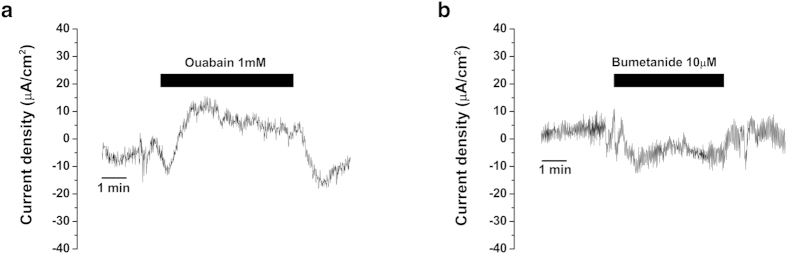
Representative figures for the changes in the trans-epithelial current from human ES after the application of blockers for Na^+^, K^+^-ATPase, and Na^+^, K^+^, 2Cl^−^ cotransporter. (**a**) Inhibition of apical cation absorption/anion secretion current after the application of Na^+^, K^+^-ATPase blocker ouabain (1 mM). (**b**) Inhibition of cation secretion/anion absorption current after the application of Na^+^, K^+^, 2Cl^−^ cotransporter blocker bumetanide (10 μM).

Ouabain (1 mM) and bumetanide (10 μM) were used to identify the involvement of Na^+^, K^+^-ATPase, and Na^+^, K^+^, 2Cl^−^-cotransporter in the electrogenic transport in human ES epithelium. Ouabain decreased the apical cation absorption/anion secretion current by 18.0 μA/cm^2^ (Fig. 4a) in one case with a type 2 basal current vector and by 4.1 μA/cm^2^ in one case with a type 3 basal current vector. In contrast, bumetanide decreased the cation secretion/anion absorption current by 11.2 μA/cm^2^ (Fig. 4b) in one case with a type 1 basal current vector and by 3.0 μA/cm^2^ in one case with a type 2 basal current vector.

These results indicate that multiple K^+^ channels, such as the Kir, Kv, K_2P_, and SK channels distributed in human ES epithelium and the trans-epithelial current was dependent on those functional K^+^ channels. Na^+^, K^+^ -ATPase and Na^+^-K^+^-2Cl^−^ cotransporter likely supplied K^+^ into the ES epithelial cells, which could be a source for K^+^ ion transport through K^+^ channels.

### Ion channels, transporters, and exchangers in human ES as identified by LC-MS/MS

LC-MS/MS was performed using human ES tissue to identify ion channels, transporters, and exchangers carrying the K^+^ ion. A total of 2762 proteins were identified from the human ES by LC-MS/MS. Among the proteins, 37 were ion channels, transporters, and exchangers (Table 1). These proteins consisted of Ca^2+^ channels, transient receptor potential (TRP) channels, K^+^ channels, Na^+^ channels, non-selective cation channels, Na^+^, K^+^-ATPase, and ion exchangers, and Cl^−^ channel. The K^+^ channels that were identified in LC-MS/MS were 3 voltage-gated channels [KCNB1(gi|186798), KCNC4(gi|338077), and KCNH1(gi|27437001)] and 1 apamin-sensitive SK channel [KCNN2 (gi|10334701)].

### RT-PCR of K^+^ channels identified by LC-MS/MS and electrophysiological/pharmacological studies

**Figure 5:**
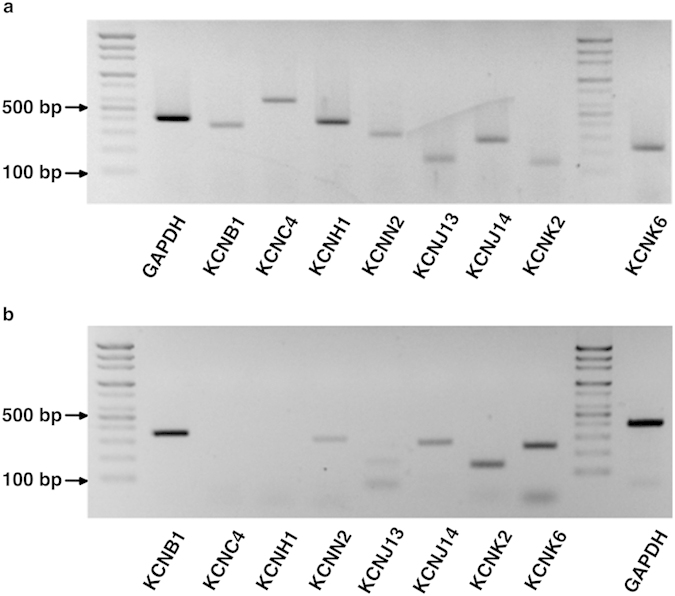
Transcript expression of the final candidate K^+^ channels (KCNB1, KCNC4, KCNH1, KCNN2, KCNJ13, KCNJ14, KCNK2, and KCNK6) identified by electrophysiological/pharmacological and LC-MS/MS experiments. (**a**) Transcript expression of the candidate K^+^ channels in the positive control (human kidney). (**b**) Transcript expression of the candidate K^+^ channels in the human ES. Transcripts of KCNB1, KCNN2, KCNJ14, KCNK2, and KCNK6 were detected by RT-PCR.

RT-PCR was performed to verify the transcript expression of K^+^ channels as identified by LC-MS/MS (KCNB1, KCNC4, KCNH1, and KCNN2) and that are hypothesized to exist according to electrophysiological and pharmacological studies (KCNJ13, KCNJ14, KCNK2, and KCNK6) in the human ES. As a result, the transcript expression of KCNB1, KCNN2, KCNJ14, KCNK2, and KCNK6 were identified by RT-PCR, whereas those of KCNC4, KCNH1, and KCNJ13 were not identified (Fig. 5). The faint double bands of KCNJ13 in a human endolymphatic sac tissue sample (Fig. 5B) were revealed to be a product of non-specific amplification at a low target concentration after the sequencing of the purified PCR product.

### Immunohistochemistry of K^+^ channels

**Figure 6:**
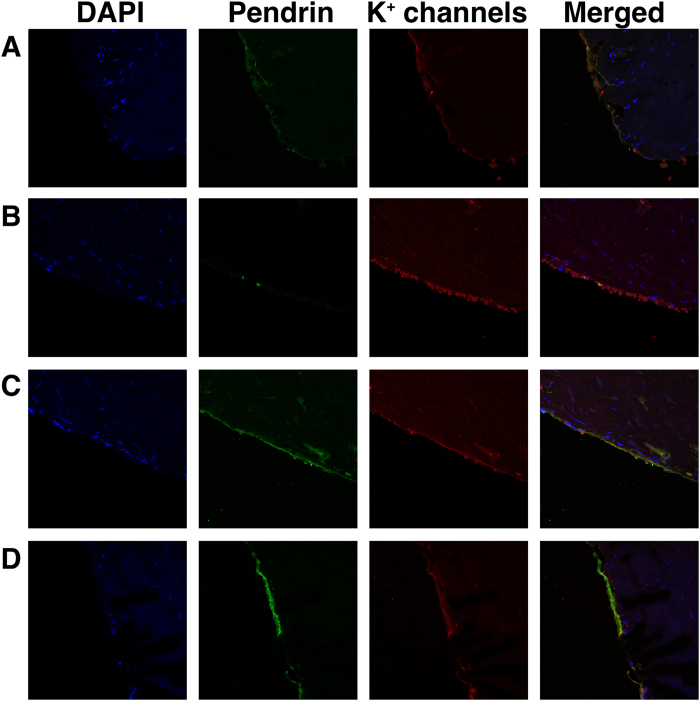
Immunofluorescent staining of K^+^ channels (KCNB1, KCNN2, KCNJ14, KCNK2, and KCNK6) identified by RT-PCR. (**a**) KCNN2. (**b**) KCNJ14. (**c**) KCNK2. (**d**) KCNK6. The K^+^ channels except for KCNB1 were detected by immunofluorescent staining. Pendrin antibody was used for the validation of the ES epithelium because pendrin exists in mitochondria-rich cells of ES epithelial cells.

Expression and localization of the K^+^ channels that were identified by RT-PCR were evaluated by immunohistochemistry. Immunohistochemical assessments indicated that all of the candidate channels except for KCNB1 were expressed in the ES epithelium (Fig. 6). These four channels were co-expressed with pendrin, which was thought to exist in the mitochondria-rich cells of ES epithelium^10^ (Fig. 6a–d), but KCNJ14 was expressed in the ES epithelium without pendrin expression (Fig. 6b), which was suspected to occur in other cell types of ES epithelium, such as ribosome-rich cells.

## Discussion

This report is the first to functionally identify electrogenic transport dependent on functional K^+^ channels in human ES epithelium. Thus far, there has been little interest in the K^+^ channel contribution in the electrogenic transport in ES epithelium; however, K^+^ ion transport–related electrogenic transport in human ES epithelium was positively identified in this study. This should contribute to the maintenance of a unique ionic milieu of the luminal fluid in human ES. The results of this study could be a basis for elucidating the role of human ES in regulating inner ear homeostasis and fluid regulation.

Evidence for the presence of K^+^ channels in the ES epithelium has been suggested by two animal studies with functional experiments^16^,^17^. The voltage-dependent outward K^+^ current was identified in the patch clamp experiment using guinea pig ES epithelium, which was blocked by externally applied 4-AP (1 mM), Ba^2+^ (5 mM), and tetraethylammonium chloride (TEA, 20 mM)^17^. The K^+^ channel was thought to exist on the basolateral surface of ES epithelial cells, where it would function in conjunction with the Na^+^, K^+^ - ATPase to maintain the driving force for cation absorption through the apical membrane of the ES epithelial cells. Indirect evidence for the apical K^+^ conductance in the ES epithelium of guinea pig was proposed by measuring changes in the ES trans-epithelial potential while applying various K^+^ channel blockers to the ES lumen^16^. ES trans-epithelial potential was reduced immediately after the injection of control solution (100 mM NaCl, 15 mM KCl, 1 mM CaCl_2_, 1 mM MgCl_2_, 60 mM mannitol; osmolarity = 280 mOsm/l) and recovered to the initial level within 30 min. However, the recovery of ES trans-epithelial potential was more delayed after the injection of TEA (20 mM), 4-AP (1 mM), and bumetanide (100 μM) compared to the control solution, which represented apical K^+^ conductance in the ES epithelium. Although these two studies suggested the location of K^+^ channels in ES epithelium, the location could not be exactly defined by the experiments. The former study inferred that a voltage-dependent K^+^ channel was located on the basolateral surface of the ES; however, the location of the K^+^ channels could not be exactly determined using only the whole cell patch clamp technique; the voltage-dependent K^+^ channel could be located on either the apical or the basolateral surface of the ES epithelium. The latter study applied the K^+^ channel blockers to the ES lumen (the apical side of the ES epithelium), which changed the recovery of ES trans-epithelial potential; therefore, the K^+^ channels could be suspected to be on the apical surface. However, this could not be used as direct evidence of apical K^+^ channel distribution, as K^+^ channel-mediated current was not directly measured on the apical surface of ES epithelium using a specific method such as an excised patch technique. In addition, the locally applied K^+^ channel blockers were able to influence the ion transport through multiple ion channels in the ES epithelium, which ultimately caused ES trans-epithelial potential change. In our study, we also could not be certain whether the location of the K^+^ channels identified by the experiments was apical or basolateral. Decreases in apical cation secretion current after the application of K^+^ channel blockers were detected and indicate two possibilities. One is the decreased K^+^ secretion through K^+^ channels in the apical side and the other is the changes in net trans-epithelial current vectors induced by the changes of various ion channel activities in ES epithelial cells mediated by K^+^ ion transport. For example, the active K^+^ channels may contribute to the trans-epithelial current change in some other way, such as by membrane voltage regulation of a Cl^−^ current.

The current changes that were induced by K^+^ channel blockers, such as Ba^2+^ and 4-AP, were significantly larger than the amount of basal trans-epithelial current of human ES epithelium, indicating that multiple other cation absorption and anion secretion events should occur through the apical surface of human ES in addition to cation secretion/anion absorption dependent on functional K^+^ channels, which is also supported by the finding that the net basal current vectors varied, such as cation secretion/anion absorption (type 1), cation absorption/anion secretion (type 2), and neutral (type 3), regardless of the response to the K^+^ channel blockers. As reported previously in animal experiments, various ion channels absorbing Na^+^ exist on the apical surface of ES epithelium^12^. In addition, several pieces of evidence support the existence of chloride channels in the apical surface of the ES, and these channels would produce, when active and with frequently-observed electrochemical gradient favourable to Cl^−^ secretion, the same directional current vector as do Na^+^-absorbing channels^1^,^18^. The activity of these channels can produce current vectors opposite to the cation secretion/anion absorption current vectors dependent on functional K^+^ channels, which finally caused three different types of net trans-epithelial current vectors according to the location of measurement, as shown in our experiment. Therefore, the amount of current change after the application of K^+^ channel blockers could be larger than the net basal current in human ES epithelium, especially with the application of non-specific broad-spectrum K^+^ channel blockers because they blocked multiple K^+^ channels, which caused a large amount of current change (the use of apamin, a specific blocker of KCNN2, induced a smaller amount of current change than the amount of basal current). The difference in the net basal current vector in each experiment was caused by the heterogeneous distribution of different epithelial cell types with unique ion channel distributions. The ion channel distribution according to each cell type should be functionally identified in future studies using molecular markers for each cell type.

We used RT-PCR and immunohistochemistry to identify the K^+^ channel candidates as revealed by LC-MS/MS and electrophysiological/pharmacological experiments. As a result, we finally identified the K^+^ channels of the ES epithelium among several candidate channels as KCNN2, KCNJ14, KCNK2, and KCNK6. The distribution of these channels in the ES epithelium was likely to be different according to cell type. Thus far, it was thought that most ion channels, including pendrin, were distributed in mitochondria-rich cells^6^,^7^,^8^,^9^,^10^. In this study, most of the K^+^ channels, such as KCNN2, KCNK2, and KCNK6, were co-expressed in the cells in which pendrin was expressed, whereas KCNJ14 was mostly expressed in the cells in which pendrin was not expressed, meaning that KCNN2, KCNK2, and KCNK6 are likely to exist in mitochondria-rich cells and that KCNJ14 can exist in cells other than mitochondria-rich cells. Therefore, we can suggest that both cell types contribute to the homeostasis of ES luminal fluid by regulating the ion transport, in which K^+^ channels necessarily participate. Although we detected functional evidence for the existence of 4-AP sensitive K^+^ channels and transcript expression of 4-AP sensitive KCNB1 by the electrophysiological/pharmacological experiments and RT-PCR, we could not identify KCNB1 at the protein level by immunohistochemistry. It was not surprising in that RNA expression did not always correlate with protein expression and function. In addition, a proteomic analysis could yield false-positive results, as this method provides screening data for the protein compositions of certain tissues by analysing the amino acid sequences using spectrometry and statistical analyses. We also could not identify inwardly rectifying K^+^ channels in the LC-MS/MS that were detected in the electrophysiological/pharmacological experiment, representing the possible limitation of LC-MS/MS as a screening test. Instead, there should be 4-AP-sensitive K^+^ channels other than KCNB1 in the human ES epithelium because 4-AP is a non-selective, wide-spectrum, voltage-dependent K^+^ channel blocker. These channels should be identified in the near future using more sensitive screening tests followed by functional and molecular studies using more specific pharmacologic agents and specific markers.

One of the main roles of K^+^ channels in the inner ear is the maintenance of high K^+^ ions in the endolymph. In addition, K^+^ channels in the cochlea are involved in creating high positive potential (endocochlear potential ≈80–100 mV) in the endolymphatic space of the cochlear compartment to facilitate the K^+^ absorption through hair cells^3^. The K^+^ ion concentration of the endolymph is maintained mainly by K^+^ secretion from inner ear epithelial cells. The two sites for K^+^ secretion are the stria vascularis in the cochlea compartment and the dark cells in the vestibular compartment^19^. The mechanism for K^+^ secretion in both structures is virtually identical. The stria vascularis consists of three cell layers (basal cells, intermediate cells, and marginal cells) and low K^+^ concentration fluid fills the space between intermediate cells and marginal cells (intrastrial space), although dark cells do not have such layers^19^. The marginal cells and basal cells are each linked among themselves by tight junctions. In the stria vascularis, K^+^ ions are taken up from the abluminal fluid (also known as the perilymph) through Na^+^, K^+^-ATPase and NKCC in basal cells and moved to intermediate cells via gap junctions^19^. Intermediate cells release the K^+^ ions into the intrastrial space through KCNJ10, and the released K^+^ ions are taken up into marginal cells through basolateral Na^+^, K^+^-ATPase and NKCC and then secreted into the endolymph through KCNQ1/KCNE1 K^+^ channels^19^. The equilibrium K^+^ potential generated by KCNJ10 in intermediated cells in conjunction with a low extracellular K^+^ concentration in the intrastrial space and a high cysosolic K^+^ concentration in intermediate cells creates the endocochlear potential^19^. In dark cells, K^+^ ions are taken up through basolateral Na^+^, K^+^-ATPase and NKCC, and then secreted into the endolymph through KCNQ1/KCNE1 K^+^ channels^19^; however, there is no high positive potential in the vestibular compartment, because of the absence of intermediate cells and the absence of completely separated low K^+^ concentration space as found in stria vascularis.

Although the K^+^ secretion and their roles were well-known in the cochlear and vestibular compartment, the distribution and the roles of K^+^ channels in ES is still unclear. The K^+^ channels in human ES varied in our study, and it seems that those channels essentially contribute to the electrogenic transport in ES epithelium. Na^+^, K^+^-ATPase and NKCC were also likely to be involved in the K^+^ ion transport by placing K^+^ ions into cytosol. Other ion channels, transporters, and exchangers responsible the movement of abundant ions, such as Na^+^, Cl^−^, and Ca^2+^ could be involved in the regulation of the electrogenic transport of ES epithelium, which should be closely related to K^+^ channel activity as various ion movements have been observed in ES epithelium in animal experiments^12^. To identify the exact mechanism involved in producing net electrogenic transport in human ES epithelium related to the K^+^ ion transport, further studies for various cation and anion movements through the ES epithelium should be conducted.

## Conclusion

Trans-epithelial current dependent on functional K^+^ channels was identified in the human ES epithelium by electrophysiological, pharmacological, and molecular biological methods in this study. K^+^ channels, including KCNN2, KCNJ14, KCNK2, and KCNK6, were involved in the electrogenic transport of human ES epithelium coupled with the activity of NKCC/Na^+^, K^+^ - ATPase. This K^+^ transport is thought to play an important role in the maintenance of inner ear homeostasis by participating in the regulation of electrogenic transport.

## Materials and Methods

### Harvesting ES

Human ES was harvested during acoustic tumour surgery via a translabyrinthine approach in 16 patients (M:F = 5:11, mean age: 50.9 ± 3.0 years). The bony surface covering the posterior fossa dura and ES was carefully thinned with drilling and then peeled off to expose the whole ES. After exposing the ES, ~200 μl of physiological saline was injected to prevent damage to the luminal surface during dissection. The ES was carefully dissected after the incision of the ES border, and the luminal surface was exposed. The harvested ES was immersed in perilymph-like solution (specified in *transepithelial current measurement*) and was used immediately for the experiment.

### Trans-epithelial current measurement

The scanning vibrating electrode technique was used to measure trans-epithelial current from the ES epithelium as previously described^20^. To measure the trans-epithelial current from the tissue, the harvested ES was divided into small pieces (~3 × 3 mm), and the prepared tissue was smoothly folded apical side-out (Fig. 2a). The tissue was mounted in a perfusion chamber of an inverted microscope (IX51, Olympus, Tokyo, Japan) and continuously perfused at 37 °C with an exchange rate of 1.2 times/sec. The current density was monitored by vibrating a platinum-iridium wire microelectrode insulated with parlene-C (Micro Electrodes, Gaithersburg, MD, USA) and coated with Pt black on the exposed tip. The electrode tip of the probe was vibrated at two frequencies between 400 and 700 Hz along horizontal (*x*) and vertical (*z*) axes by piezo-electric bimorph elements (Applicable Electronics, Forestdale, MA, USA) and was positioned 5 ± 2 μm from the apical surface of the epithelium (Fig. 2a). The *x*-axis was perpendicular to the face of the epithelium. A platinum-black electrode served as reference in the bath chamber. The signals from the oscillators driving the probe, which were connected to a dual-channel phase-sensitive detector (Applicable Electronics), were digitized (16 bit) at a rate of 0.5 Hz. The electrode was positioned so that the current density showed a maximum *x* value and a minimum *z* value. The data that were derived from the x direction current density were plotted with Origin software, version 8.0. (OriginLab Software, Northampton, MA, USA).

For electrophysiological experiments, a perilymph-like physiological saline solution [150 mM NaCl, 3.6 mM KCl, 1 mM MgCl_2_, 0.7 mM CaCl_2_, 5 mM glucose, and 10 mM HEPES (pH 7.4)] was used for perfusion. The pharmacologic agents that were used in this study were purchased from Sigma (St. Louis, MO, USA). All agents except ouabain and bumetanide were dissolved in the perilymph-like solution before application. Ouabain and bumetanide were dissolved in DMSO (Sigma) and then diluted to <0.1% DMSO in perilymph-like solution before application. DMSO at this concentration had no effect on current density^21^.

### Identification of the ES protein by LC-MS/MS

For the screening of the existence of various ion channels, transporters, and exchangers in human ES, LC-MS/MS was performed. Two harvested ES tissues were suspended in 500 μl of sample buffer (7 M urea, 2 M thiourea, 4% CHAPS, 0.5% ampholyte, 100 mM DTT, 40 mM Tris, 0.002% bromophenol blue) and were sonicated for 10 seconds 10 times with 10-second intervals. Then, 10 μl of DNase (10,000 U/ml) was added and incubated at 4 °C for 30 min. After incubation, the supernatant was centrifuged at 105,000 g for 45 min. Then, 50% trichloroacetic acid was added to a final concentration of 5–8%, and the sample was incubated on ice for 2 hours. The sample was centrifuged at 14,000 g for 15 min, and the supernatant was discarded. Cold acetone (200 μl) was added, and the sample was incubated on ice for 15 min. The sample was centrifuged at 14,000 g for 20 min, and the supernatant acetone was discarded. The pellet was dried in air and dissolved in sample buffer. The protein concentration was quantified by the Bradford protein assay.

Thirty micrograms of ES protein was used for one-dimensional gel electrophoresis (1-DE) . The samples were lyophilized and dissolved in 15 μl of distilled water. Samples were subjected to sodium dodecyl sulphate gel electrophoresis on an 8–16% Tris/Glycine gel and stained with Coomassie Brilliant Blue. The entire 1-DE gel lane was cut into 8 pieces according to molecular weight for digestion. After reduction with dithiothreitol and alkylation with iodoacetamide, each piece of gel was treated with trypsin for *in situ* digestion and then washed with 10 mM ammonium bicarbonate and 50% acetonitrile and swollen in digestion buffer containing 50 mM ammonium bicarbonate, 5 mM CaCl2, and 1 μg of trypsin. Next, the gel was incubated at 37 °C for 12 hours. Peptides were recovered over the course of 2 extraction cycles with 50 mM ammonium bicarbonate and 100% acetonitrile. The resulting peptide extracts were pooled, lyophilized, and stored at -20 °C.

Nano LC-MS/MS analysis was performed on an Agilent 1100 Series nano-LC and linear trap quadrupole (LTQ)-mass spectrometer (Thermo Electron, San Jose, CA). The capillary column that was used for LC-MS/MS analysis (150 mm × 0.075 mm) was obtained from Proxeon (Odense M, Denmark), and slurry-packed in-house with 5 μg, 100 Å pore size Magic C18 stationary phase (Michrom Bioresources, Auburn, CA). Mobile phase A for LC separation was 0.1% formic acid in deionized water, and mobile phase B was 0.1% formic acid in acetonitrile. Chromatography was performed using a linear gradient from 5% B to 35% B over 100 min, from 40% B to 60% B over 10 min, and from 60% B to 80% B over 20 min. The flow rate was maintained at 300 nl/min after splitting. Mass spectra were acquired using data-dependent acquisition with a full mass scan (400–1800 m/z) followed by MS/MS scans. Each acquired MS/MS scan represented the average of one microscan on the LTQ. The temperature of the ion transfer tube was controlled at 200 °C, and the spray was 1.5–2.0 kV. The normalized collision energy was set at 35% for MS/MS.

The MASCOT and SEQUEST (Bioworks software version 3.2, Thermo Electron, Waltham, MA, USA) search engines were used to search the UniProt human protein databases (release 14.8; 82728 sequences) for tandem mass spectra. Mass tolerances of 1.2 and 0.6 Da were used for precursor and fragment ions, respectively. The search included variable modifications of oxidation on methionine and carbamidomethyl of cysteine. PeptideProphet and ProteinProphet were used to estimate the false discovery rate (FDR) for any minimum probability that was used as a cut-off for the MASCOT and SEQUEST search results. Proteins for ion channels, transporters, and exchangers in the data were checked.

### RT-PCR

After the homogenization of the harvested human ES tissue, the total RNA was extracted using TRIzol® (Invitrogen, Carlsbad, CA, USA) following the manufacturer’s protocol. The quantity and quality of isolated RNA were determined with a NanoDrop ND-100 spectrophotometer (NanoDrop Technologies, Wilmington, DE, USA) and by analysing the 18S and 28S rRNA bands after electrophoresis. cDNA was synthesized from 3 μg of total RNA with random hexamer primers (Perkin Elmer Life Sciences, Boston, MA, USA and Roche Applied Science, Mannheim, Germany), AMV reverse transcriptase (Perkin Elmer Life Sciences), and RNase inhibitor (Perkin Elmer Life Sciences). The reverse transcription step was performed for 10 min at room temperature, 30 min at 50 °C, and 15 min at 95 °C.

To verify the genomic expression of ion channels, transporters, and exchangers that were identified by LC-MS/MS and electrophysiological/pharmacological studies, RT-PCR was performed. The transcript of each ion channel was amplified using gene-specific primers (Table 2). The PCR conditions included 30 cycles of denaturation at 94 °C for 30 seconds, annealing at 54 °C for 30 seconds, and polymerization at 72 °C for 30 seconds. The PCR products were run on a 1.5% agarose gel and visualized with ethidium bromide under a transilluminator. Total RNA extracted from human kidney (Clontech, Takara Korea Biomedical Inc., Seoul, Korea) was used as a positive control for K^+^ channels. All PCR products were purified by a PCR purification kit (Qiagen, Valencia, CA, USA), and the resulting purified PCR products were sequenced to verify the identity of the RT-PCR product.

### Immunohistochemistry

To verify the protein expression of K^+^ channels as identified by LC-MS/MS, RT-PCR and electrophysiological/pharmacological studies, immunohistochemistry was performed. We used pendrin as a marker for the mitochondria-rich cells of the ES. Human ES tissues were fixed with 4% paraformaldehyde for 24 hours and then dehydrated and embedded in paraffin. Paraffin blocks were sectioned into 5-μm-thick slices and attached on slide glasses. The samples were deparaffinized twice in xylene for 5 min and hydrated with 100% and 95% ethanol twice for 3 and 2 min, respectively. After washing the sample with distilled water, antigen retrieval was performed in pH 9.0 Tris-EDTA buffer for 20 min using a microwave. The samples were blocked with blocking solution (Lab Vision Ultra V Block, Thermo Scientific, Waltham, MA, USA) for 1 hour and then washed for 5 min 3 times in PBS. The samples were incubated overnight at 4 °C with the first primary antibodies [KCNB1 (1:100, ab111122, Abcam, Cambridge, MA, USA), KCNN2 (1:200, LS-A9578, LSBIO, Seattle, WA, USA), KCNK2 (1:200, ab83932, Abcam), KCNK6 (1:200, hpa040184, Atlas Antibodies, Stockholm, Sweden), and KCNJ14 (1:50, sc-23633, Santa Cruz, Dallas, TX, USA) for each sample]. After incubation, the samples were washed and incubated with secondary antibodies according to the use of first primary antibodies [1:200 goat anti-rabbit Alexa Fluor 568 for KCNB1, KCNN2, KCNK2, KCNK6 (ab175471, Abcam) or 1:200 donkey anti-goat Alexa Fluor 488 for KCNJ14 ab175704, Abcam)] for 1 hour at room temperature. The samples were washed and blocked with the blocking solution for 1 hour. Then, the samples were incubated overnight at 4 °C with the second primary antibody (pendrin, 1:50, sc-23779, Santa Cruz). After incubation, the samples were washed and incubated with secondary antibodies for pendrin [1:200 donkey anti-goat Alexa 488 Fluor (ab150129, Abcam) for the samples previously treated with Alexa 568 Fluor and 1:200 donkey anti-goat Alexa 568 (ab175704) for the samples previously treated with Alexa 488 Fluor] for 1 hour at room temperature. The samples were washed, and the nuclei of the tissues were stained with 4’,6-diamidino-2-phenylindole (DAPI). The samples on the slide glasses were mounted with cover glass, and the expression levels of the channels were observed with confocal laser scanning microscopy (LSM780, Carl Zeiss, Jena, Germany)

### Data analysis

The results are presented as the means ± SE from *n* observations. The amount of the trans-epithelial current change was calculated as follows. The basal current amount was calculated by subtracting the current at the reference point (400 μM from the epithelium) from the trans-epithelial current obtained during the first 30 seconds after locating the probe on the apical side of the epithelium. The current changes that were induced by the application of pharmacologic agents were calculated by subtracting the basal current from the trans-epithelial current obtained during the last 30 seconds after the application of the pharmacologic agent. The significance of the changes of the current density after the application of pharmacologic agents was calculated using the student t-test or Mann-Whitney U test. A value of *p* < 0.05 was considered significant.

### Ethics statement

This study was approved by the institutional review board of Severance Hospital, Yonsei University College of Medicine, and written informed consent was obtained from each of the participants. The experimental methods were carried out in accordance with the approved guidelines.

## Additional Information

**How to cite this article**: Kim, S. H. *et al.* Electrogenic transport and K^+^ ion channel expression by the human endolymphatic sac epithelium. *Sci. Rep.* **5**, 18110; doi: 10.1038/srep18110 (2015).

## Figures and Tables

**Figure 1:**
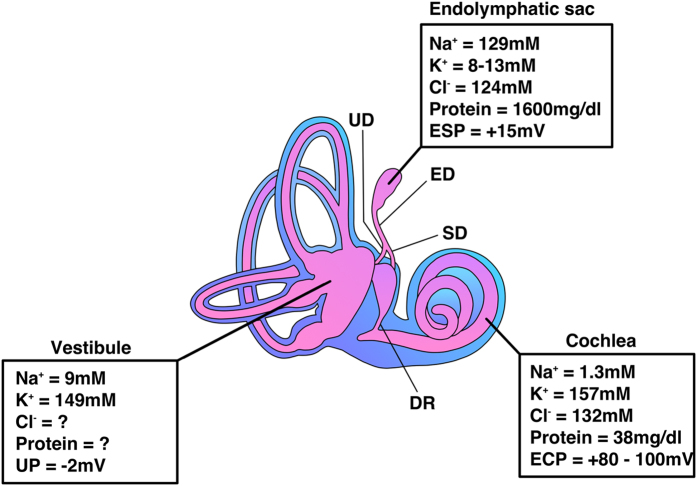
Structures and chemical compositions of endolymph of the inner ear. The endolymphatic sac is connected to the cochlear and vestibular systems through the ductus reuniens (DR), the utricular duct (UD), and the saccular duct (SD) and has a different endolymphatic potential and ion and protein composition compared to those of the cochlear and vestibular systems. ESP, endolymphatic sac potential; ECP, endocochlear potential; UP, utricular potential.

**Table 1:** Ion channels, transporters, and exchangers that were identified in the human ES by LC-MS/MS.

Accession number	Description (gene name)	Max coverage (%)	Max protein score
gi|6090615	dihydropyridine receptor alpha 2 subunit (CACNA2D1)	13.7	83
gi|38505268	voltage-dependent T-type calcium channel subunit alpha-1G isoform 14 (CACNA1G)	10.5	72
gi|7630181	alpha1A-voltage-dependent calcium channel (CACNA1A)	3.6	70
gi|292275	L-type voltage-dependent calcium channel (CACNA1)	6.1	66
gi|179764	calcium channel alpha-1D subunit (CACNA1D)	6.2	64
gi|20070163	voltage-dependent T-type calcium channel subunit alpha-1G isoform 1 (CACNA1G)	8.5	63
gi|5565888	T-type calcium channel alpha1I subunit (CACNA1I)	4.7	56
gi|14336738	voltage dependent t-type calcium channel alpha-1H subunit (CACNA1H)	5.4	52
gi|443761	voltage-operated calcium channel; alpha-1 subunit (CACNA1E)	7.2	54
gi|7159257	calcium channel alpha1E subunit; delta39 splice variant (CACNA1E)	10	56
gi|9711929	alpha1A-voltage-dependent calcium channel (CACNA1A)	10.3	55
gi|3183953	L-type calcium channel alpha-1 subunit (CACNA1F)	3.7	54
gi|55962840	calcium channel; voltage-dependent; N type; alpha 1B subunit (CACNA1B)	5.3	51
gi|4336152	low-voltage activated calcium channel alpha (CACNA1H)	4.9	51
gi|179762	calcium channel alpha-2b subunit (CACNA2D1)	7.1	51
gi|495868	voltage-dependent calcium channel alpha-1E-1 (CACNA1E)	5.6	50
gi|62088514	calcium channel; voltage-dependent; L type; alpha 1B subunit variant (CACNA1B)	11	46
gi|21314671	transient receptor potential cation channel subfamily M member 4 isoform 1 (TRPM4)	15.8	52
gi|54632969	long transient receptor potential channel 3 (TRPM3)	3.3	52
gi|76780233	TRPC4 protein (TRPC4)	5.4	54
gi|194733735	short transient receptor potential channel 3 isoform a (TRPC3)	7.9	47
gi|24119274	sodium leak channel non-selective protein (NALCN)	8.9	65
gi|189047	Na^+^ channel (SCN7A)	3.1	59
gi|25014054	RecName: Full = Sodium channel protein type 3 subunit alpha; AltName: Full = Sodium channel protein brain III subunit alpha; (AltName: Full = Sodium channel protein type III subunit alpha; AltName: Full = Voltage-gated sodium channel subtype III; AltName: Fu (SCN3A)	4.8	45
gi|6693697	voltage-gated sodium channel alpha subunit SCN12A (SCN12A)	6.9	45
gi|10334701	apamin-sensitive small-conductance Ca^2+^-activated potassium channel (KCNN2)	7.4	57
gi|186798	voltage-gated potassium channel (KCNB1)	10.1	54
gi|338077	potassium channel protein (KCNC4)	6.4	53
gi|27437001	potassium voltage-gated channel subfamily H member 1 isoform 1 (KCNH1)	10	49
gi|21361181	sodium/potassium-transporting ATPase subunit alpha-1 isoform a (ATP1A1)	9.1	66
gi|497763	Na^+^; K+ -ATPase catalytic subunit (ATP1A3)	5.4	45
gi|14286105	RecName: Full = Plasma membrane calcium-transporting ATPase 4; Short = PMCA4; AltName: Full = Matrix-remodelling-associated protein 1; AltName: Full = Plasma membrane calcium ATPase isoform 4; AltName: Full = Plasma membrane calcium pump isoform 4 (ATP2B4)	14.9	53
gi|118498343	calcium-transporting ATPase type 2C member 2 (ATP2C2)	13	48
gi|17865805	sodium/calcium exchanger 3 isoform B precursor (SLC8A3)	5.3	50
gi|15147254	sodium/calcium exchanger 3 isoform A precursor (SLC8A3)	9	46
gi|4505697	Pendrin (SLC26A4)	6	44
gi|68534512	Anoctamin 6 (ANO6)	8.7	52

Proteins searched through MASCOT (version 2.2.07) against the NCBInr database. The search parameters were: 1) Enzyme specificity - Trypsin; 2) Maximum missed cleavages - 2; 3) Carbamidomethyl (C); Oxidation (M) as variable modifications.

**Table 2:** Primer information for RT-PCR of K^+^ channels.

Gene	GenBank Accession No.	Forward primer	Reverse primer	Amplicon size, bp
KCNB1	XM_006723784.1	ACCGAATCCAACAAGAGCGT	CAGCAGAGTCCCCCAACAAT	356
KCNC4	NM_001039574.2	CTCGGGGTAAGTACCACACC	TCTTGGCAGGTCTCTTTCCG	598
KCNH1	NM_172362.2	GGTAGGTCCTGTCCCTGTCA	TGCTAAATGGGCTCAGCTTT	389
KCNN2	NM_170775.2	ACACTTTGGTGGACTTGGCA	AGGTTGGTGGTGCTGTGGAA	294
KCNJ13	NM_001172416.1	CACTTCAAATGGATGGCGCT	CCACAAAAGCACCAGAGCAC	154
KCNJ14	NM_013348.3	CTGGTGGTCATTCTCGAGGG	AGAGCCGGGGAAACTAGACT	258
KCNK2	NM_014217.3	GGTGGGAGAGTTCAGAGCAC	GAGAGCTTCCGCTTGATGGA	135
KCNK6	NM_004823.1	GGTGCTGGTCACAGTCTACC	TACCTGGGGATGGAAGCGTA	237

## References

[b1] Couloigner V., Loiseau A., Sterkers O., Amiel C. & Ferrary E. Effect of locally applied drugs on the endolymphatic sac potential. Laryngoscope. 108, 592–598 (1998). 9546276 10.1097/00005537-199804000-00024

[b2] Kim S. H. *et al.* Albumin-like protein is the major protein constituent of luminal fluid in the human endolymphatic sac. PLoS One. 6, e21656 (2011). 21738753 10.1371/journal.pone.0021656 PMC3126852

[b3] Marcus D. C. & Wangemann P. Inner ear fluid homeostasis in *The ear* (ed. Fuchs P. A.) 213–230 (Oxford University Press, 2010).

[b4] Nakaya K. *et al.* Lack of pendrin HCO3- transport elevates vestibular endolymphatic [Ca2+] by inhibition of acid-sensitive TRPV5 and TRPV6 channels. Am J Physio Renal physiol. 292, F1314–1321 (2007). 10.1152/ajprenal.00432.2006 PMC2515270 17200157

[b5] Barbara M., Rask-Andersen H. & Bagger-Sjoback D. Ultrastructure of the endolymphatic sac in the mongolian gerbil. Arch Otorhinolaryngol. 244, 284–287 (1987). 3439917 10.1007/BF00468637

[b6] Dahlmann A. & von During M. The endolymphatic duct and sac of the rat: a histological, ultrastructural, and immunocytochemical investigation. Cell Tissue Res. 282, 277–289 (1995). 8565057 10.1007/BF00319118

[b7] Hultcrantz M. & Schindler R. A. Murine endolymphatic sac development in tissue culture: an *in vitro* model for sac function. Acta Otolaryngol. 109, 245–255 (1990). 2316347 10.3109/00016489009107440

[b8] Lee H. J. *et al.* Expression of anion exchangers in cultured human endolymphatic sac epithelia. Otol Neurotol. 33, 1664–1671 (2012). 23007642 10.1097/MAO.0b013e31826bf3d3

[b9] Lundquist P. G. Aspects on endolymphatic sac morphology and function. Arch Otorhinolaryngol. 212, 231–240 (1976). 990076 10.1007/BF00453671

[b10] Peters T. A., Tonnaer E. L., Kuijpers W., Cremers C. W. & Curfs J. H. Differences in endolymphatic sac mitochondria-rich cells indicate specific functions. Laryngoscope. 112, 534–541 (2002). 12148867 10.1097/00005537-200203000-00023

[b11] Kimura R. S. Experimental blockage of the endolymphatic duct and sac and its effect on the inner ear of the guinea pig. A study on endolymphatic hydrops. Ann Otol Rhinol Laryngol. 76, 664–687 (1967). 6046009 10.1177/000348946707600311

[b12] Miyashita T. *et al.* Presence of FXYD6 in the endolymphatic sac epithelia. Neurosci lett. 513, 47–50 (2012). 22343024 10.1016/j.neulet.2012.02.005

[b13] Bagger-Sjoback D., Friberg U. & Rask-Anderson H. The human endolymphatic sac. An ultrastructural study. Arch Otolaryngol. 112, 398–409 (1986). 10.1001/archotol.1986.03780040038008 3947460

[b14] Goldstein S. A. *et al.* International Union of Pharmacology. LV. Nomenclature and molecular relationships of two-P potassium channels. Pharmacol Rev. 57, 527–540 (2005). 16382106 10.1124/pr.57.4.12

[b15] Kubo Y. *et al.* International Union of Pharmacology. LIV. Nomenclature and molecular relationships of inwardly rectifying potassium channels. Pharmacol Rev. 57, 509–526 (2005). 16382105 10.1124/pr.57.4.11

[b16] Teixeira M. *et al.* Evidence for apical K conductance and Na-K-2Cl cotransport in the endolymphatic sac of guinea pig. Hea Res. 128, 45–50 (1999). 10.1016/s0378-5955(98)00197-x 10082282

[b17] Wu D. & Mori N. Outward K+ current in epithelial cells isolated from intermediate portion of endolymphatic sac of guinea pigs. Am J Physiol. 271, C1765–1773 (1996). 8944662 10.1152/ajpcell.1996.271.5.C1765

[b18] Matsubara A., Miyashita T., Inamoto R., Hoshikawa H. & Mori N. Cystic fibrosis transmembrane conductance regulator in the endolymphatic sac of the rat. Auris Nasus Larynx 41, 409–412 (2014). 24598307 10.1016/j.anl.2014.02.005

[b19] Zdebik A. A., Wangemann P. & Jentsch T. J. Potassium ion movement in the inner ear: insights from genetic disease and mouse models. Physiology. 24, 307–316 (2009). 19815857 10.1152/physiol.00018.2009 PMC4415853

[b20] Kim B. G. *et al.* Developmental changes of ENaC expression and function in the inner ear of pendrin knock-out mice as a perspective on the development of endolymphatic hydrops. PLoS One. 9, e95730 (2014). 24752462 10.1371/journal.pone.0095730 PMC3994121

[b21] Pondugula S. R. *et al.* Glucocorticoid regulation of genes in the amiloride-sensitive sodium transport pathway by semicircular canal duct epithelium of neonatal rat. Physiol Genomics. 24, 114–123 (2006). 16263802 10.1152/physiolgenomics.00006.2005

